# Gut Microbiota in Dholes During Estrus

**DOI:** 10.3389/fmicb.2020.575731

**Published:** 2020-11-30

**Authors:** Xiaoyang Wu, Yongquan Shang, Qinguo Wei, Jun Chen, Huanxin Zhang, Yao Chen, Xiaodong Gao, Zhiyong Wang, Honghai Zhang

**Affiliations:** ^1^College of Life Sciences, Qufu Normal University, Qufu, China; ^2^College of Marine Life Sciences, Ocean University of China, Qingdao, China; ^3^Shijiazhuang Wildlife Conservation Center, Shijiazhuang, China

**Keywords:** dhole, *Cuon alpinus*, metagenomic, host microbiota interactions, estrus, gut microbiota, fecal microbes

## Abstract

The co-evolution of gut microbes and the host plays a vital role in the survival and reproduction of the host. The dhole (*Cuon alpinus*) has been listed as endangered species by the International Union for Conservation of Nature; therefore, conservation and effective breeding of dholes are essential. Effective estrus can promote reproduction. However, little is known about the relative contribution of estrus in shaping the structure and the functions of fecal microbiota. Here, we investigated the potential association between estrus and the fecal microbiota in dholes using shotgun metagenomic sequencing. We found that the estrus stages in dholes vary significantly in terms of gut bacterial composition and microbiome metabolism and function. Compared with that of non-estrus, adult dholes, the microbiome of estrus adult dholes had a significantly higher abundance of *Bacillus faecalis* and *Veillonella*, which play a key role in the synthesis of sex hormones and nucleic acids, energy production, and reproductive cell division. The insulin and energy metabolism-related pathways are significantly enhanced in the gut microbes and the related gluconeogenic enzymes are significantly enriched during estrus. These findings suggest that the structure and metagenome of the fecal microbiome during estrus have a significant effect in promoting estrus in dholes, thus providing a new perspective for dhole conservation.

## Introduction

The fecal microbiota, including bacteria, fungus, and other microorganisms, plays a critical role in the maintenance of host health and disease resistance by facilitating numerous biological processes (Sekirov et al., 2010; Lynch and Hsiao, 2019). The structure of the fecal microbiome is influenced by the genome, sex, living environment, and diseases of the host (Stephens et al., 2016; Chen et al., 2017; Wu et al., 2017; Zhu et al., 2018). Interestingly, a recent study has revealed that the fecal microbiome is closely associated with emotion and behavior in a rodent model (Tillisch et al., 2017). Studies on humans revealed that many factors, including the reflection produced by the brain and physiological phenomena occurring in the body, affect the fecal microbiome, and that the fecal microbiome responds to these influences through special structural changes (Valles-Colomer et al., 2019).

Reproduction is essential for the survival of species, and effective estrus can promote reproduction (Andersson, 2016). Animals in estrus have been known to display altered behavior (Schino and Visalberghi, 2011). Physiologically, they also exhibit behaviors that are different from others (Dhami et al., 2015). The estrus in domestic animals has been effectively induced using exogenous sex hormones (estrogen and triad hormone) (Hua et al., 2004), however, these may disrupt normal hormone secretion and cause endocrine disorders and thus, cannot stably and effectively increase the mating or reproduction rate (Gonzales, 2003). The same holds true for wild animals. Additionally, wild animals have strong excitability and handling for administration of hormones may alter their behavioral responses as well expose human to zoonotic pathogens.

Colonization and reproduction of some microorganisms in the intestine can be achieved by regulating diet (Shepherd et al., 2018). If so, feeding animals an estrus-promoting diet from a distance could be a means to achieve the non-injurious protection of wild animal breeding while maintaining a safe distance from them. However, little is known about the relationship between the fecal microbiome and the estrus period.

To address these questions, we focused on the dhole (*Cuon alpinus*), a native species in the East, Southeast, and Central Asia belonging to the family *Canidae* under the order Carnivora. Dholes are classified as endangered by the International Union for Conservation of Nature (IUCN) in 2015 (Gaubert and Do Linh San, 2015). It is estimated that 75% of their historical distribution has disappeared, and that approximately 2,500 mature dholes remain in the wild. However, only less than 1,000 have reproductive capacity. Therefore, their conservation is imperative and calls for strategies to ensure reproductive and mating success, viability of offspring and their subsequent survival. Estrus and birthing seasons of these animals are influenced by natural selection and must be optimized for the survival of the species.

In this study, we collected 21 fresh fecal samples from cubs, non-estrus adults, and estrus adults and investigated the fecal microbiome of dholes in estrus in different physiological periods and environments. To our knowledge, this is the first large-scale metagenome sequencing study of dhole fecal microbiota. Our findings provide a theoretical basis for the biological mechanisms relevant to the protection of this endangered species.

## Materials and Methods

### Animals

We observed dholes housed in the Hebei Wildlife Conservation Center (Shijiazhuang, Hebei), located in a hilly area with good vegetation coverage. The habitat contains natural tree and weed species found in jungle environments, which is similar to the habitat of dholes in the wild. Dholes are highly excitable, especially during estrus. Therefore, in order to more accurately collect estrus-related information on these animals, we aimed to minimize intrusiveness into their natural lives, and to avoid interfering with their normal estrus status. The collection of fresh samples from dholes during estrus was carried out by professional breeders who housed and observed dholes for a long time. All animal experiment procedures were approved by and conducted in accordance with the ethical standards of the Qufu Normal University Animal Care and Use Committee (Permit Number: QFNU2017-002).

### Sample Collection and DNA Extraction

Fresh feces (21 samples) from captive-born dholes, including 15 from Hebei Wildlife Conservation Center and 6 from Jiangxi Wildlife Conservation Center, were collected in 2016 (Supplementary Table S1). Dholes defecate mostly in the morning but may defecate at other times, so the fecal collection time was not fixed. Our sample collection time was performed to align with the estrus period of the dhole that ranges from 5 to 7 days. After collection, the fresh fecal samples were selected for subsequent metagenomic sequencing. Based on estrus state, age, and living environment, the samples were divided into four groups: CH1, ≤1-year old; CH2, between 2 and 6 years old in non-estrus; CH3, between 2 and 6 years old in estrus; and CJ1: between 2 and 6 years old in non-estrus. The dholes in CH1, CH2, and CH3 were born and housed in Hebei, whereas those in CJ1 were from Jiangxi. The dholes of the CH1 and CH2 groups were in the same living environment and in the non-estrus period to explore the influence of age on the intestinal microbes of the dholes. The dholes of CH2 and CH3 groups were in the same living environment with similar ages, and were in estrus and non-estrus periods, respectively, to compare the effects of estrus on the intestinal microbes of dholes. The CH2 and CJ1 dholes were similar in age, were in non-estrus periods, and from different climatic environments to compare the impact of climatic environments on gut microbes. The diets of all dholes contained beef, raw eggs, chicken, and small amounts of plant-based foods formulated to be nutritionally complete. The ingestion and health status of each dhole were monitored daily by veterinarians and professional dhole keepers. All dholes were healthy and were not injected with any antibiotics or antiphlogistic drugs for the past 2 months.

To preserve the integrity of the microbiota, fresh fecal samples were collected and placed in sterile containers immediately after defecation. All samples were snap-frozen with dry ice or dipped in anhydrous ethanol (Fouhy et al., 2015) and were stored at –80°C until DNA extraction. Metagenomic DNA was extracted using a Qiagen QIAamp DNA Stool Mini Kit (Qiagen, Germany) according to the manufacturer’s protocol. The extracted DNA was stored at –20°C until further use.

### Library Construction and Metagenomic Sequencing

A total amount of 50 ng/μL DNA per sample was used for the construction of metagenomic DNA libraries. Extracted DNA was analyzed using agarose gel electrophoresis to determine its purity and integrity and quantified using Qubit^®^ 2.0 Fluorometer (Life Technologies, CA, United States). The qualified DNA was randomly fragmented by the Ovaris ultrasonic crushing instrument to a size of 350 bp. Then, the DNA fragments were end-polished, A-tailed, and ligated with the full-length adapter for sequencing, followed by PCR amplification. After the construction of the sequencing library, preliminary quantification was performed using Qubit2.0, and the library was diluted to 2 ng/μL. Then, the insert size of the library was identified using Agilent 2100 (Integrated Sciences, AUS). If the insert size was as expected, the effective concentration of the library was determined using qPCR (Real-time quantitative PCR). Accurate quantification (effective library concentration > 3 nM) can ensure the quality of the library. Metagenomic sequencing was performed on the Illumina HiSeq sequencing platform (Novogene, Tianjin, China).

### Metagenome Assembly and Construction of the Gene Catalog

The raw data were preprocessed using Readfq (V8)^1^, and we acquired a clean data for the subsequent analyses. The parameters (Luo et al., 2012) were as follows: identity ≥90%, −l 30, −v 7, −M 4, −m 200, −×400. All the procedures were conducted as previously described (Blankenberg et al., 2010). We removed the reads that matched with the dhole reference genome^2^ using SoapAligner software (Law et al., 2013). Metagenome assembly included a single sample (Luo et al., 2012; Scher et al., 2013; Qin et al., 2014; Brum et al., 2015) and mixed assemblies (Li et al., 2014; Qin et al., 2014; Sunagawa et al., 2015; Zeller et al., 2015) using the SOAPdenovo software. The open reading frames (ORF) were predicted using the MetaGeneMark software, and those shorter than 100 nt were filtered (Qin et al., 2010, 2014; Li et al., 2014; Nielsen et al., 2014; Zeller et al., 2015) from the predicted results using default parameters. We used CD-HIT software (Li and Godzik, 2006; Fu et al., 2012) to reduce redundancy and obtain a unique initial gene catalog with the following parameters: −c 0.95, −G 0, −aS 0.9, −g 1, −d 0. The clean data of each sample was mapped to the initial gene catalog using SoapAligner (soap 2.21). We calculated the reads mapped in each sample and filtered the genes with ≤2 reads (Qin et al., 2012; Li et al., 2014) in each sample. The gene catalog Unigenes was used for subsequent analysis.

### Taxonomic, Functional Assignment of Genes, and Statistical Analysis

To compare the metagenomes based on whole metagenomic sequencing data with alignment-free methods, Mash (Ondov et al., 2016) pairwise comparisons were plotted using sourmash^3^. We performed our analyses with *k* = 21. Among the values, 21, the default parameter of Mash, has been reported to be at the inflection point where the k-mer matches move from random to a representative of the read content and is generally resilient to sequencing errors and variations (Choi et al., 2018; Dong et al., 2020). Given a *k* value, Mash was run with default parameters. Once the calculated distance data were available, a phylogenetic tree was constructed using the neighbor-joining (NJ) methods to illustrate the results.

For taxonomy annotation, we blasted the unigenes against the sequences of bacteria, fungi, archaea, and viruses from the NCBI NR database using DIAMOND (Li et al., 2014) (V0.7.9)^4^. *F*- and *T*-tests are used to identify species differences at the phylum level. For comparative analysis of differences at the genus level, we chose a metastat test. We chose 0.05 as the threshold of difference significance. We selected the decrease-dimension analysis based on the detrended correspondence analysis (DCA). The reasonable decrease-dimension of the PCA (Avershina et al., 2013) (R ade4 package v 2.15.3) and NMDS (Noval Rivas et al., 2013) (R vegan package v 2.15.3), and the ANOSIM analysis (Oksanen, 2010) (R vegan package, Version 2.3), were constructed using R. Metastat (using Benjamini and Hochberg False Discovery’s Rate to correct and determine the *p*-value) and LEfSe analysis (using a default LDA score of 4) were used to identify the different species among the four groups.

For the functional annotation of genes, we compared the unigenes against a public functional database [KEGG functional database (Kanehisa et al., 2006, 2014): Version 201609^5^; CAZy database (Cantarel et al., 2009): v 20150704^6^ ] using DIAMOND software (Li et al., 2014). Based on the functional annotation result and gene abundances, the gene number table of each sample at each functional level was obtained. The gene number of a function in a sample equals the gene number annotated to this function with a non-zero abundance. Based on the abundance table of each taxonomic hierarchy and the counting of annotated gene numbers, we constructed the general relative abundance situation, abundance cluster heat map, and the decrease-dimension analysis of PCA, NMDS, and PCoA based on the Bray-Curtis distance. We also conducted the ANOSIM analysis of the difference between the groups (inside) based on functional abundance, the comparative analysis of metabolic pathways, and the Metastat and LEfSe analyses of the functional difference between groups. All visualization was conducted in the R platform.

## Results

### The Dhole in Estrus

Dholes breed based on the family unit and remain monogamous. They reach sexual maturity after their first year, and breeding occurs between their second and third years. The breeding staff of the Hebei Wildlife Conservation Center practices pair breeding after sexual maturity to avoid inbreeding and to encourage mixed raising after birth. The relationship in the previous groups is very stable. The estrus period of dholes usually occurs from December to January and last for 5–7 days. During this period, the male and female dholes become excited, scream at and chase each other, lick their genitals, and show appetite loss.

### Metagenome Preprocessing and Assembly

**FIGURE 1:**
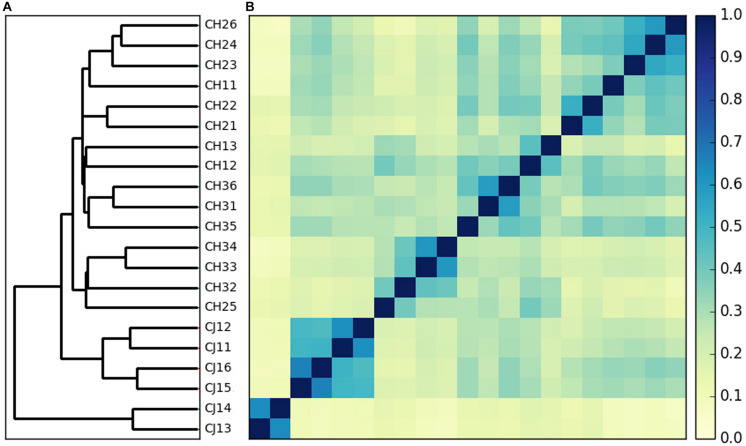
Whole metagenome comparisons of samples using Mash. **(A)** Phylogenetic tree constructed using the neighbor-joining method based on the distance calculated with Mash (s = 10,000, *k* = 21). **(B)** Heatmap based on the distance calculated by Mash (s = 10,000, *k* = 21).

Each metagenome contained an average of 6.53 Gb reads (ranging from 6.0 to 7.1 Gb), resulting in a total of 137 Gb from all samples (Supplementary Table S2). To obtain the highest data and ensure the accuracy and reliability of the downstream analysis, we preprocessed the raw data and obtained approximately 136 Gb of clean data, i.e., without low-quality reads, adapter, or host contaminants. The Q30 values of the clean data were all greater than 91%, and the average efficiency was more than 99.6%. To obtain clearer clustering results using all the metagenomic information, a comparable Mash-based tree was generated from whole genomes using a sketch size of *s* = 10,000 and *k*-mer size of *k* = 21 (Figure 1). For the metagenome dataset of dholes, the alignment-free algorithms could cluster most samples by groups. Thus, the samples from dholes in estrus and non-estrus periods were clustered into two groups despite the presence of one sporadic data point, while samples from different environments formed two clusters.

### Metagenomic Gene Prediction and Abundance Distribution

**FIGURE 2:**
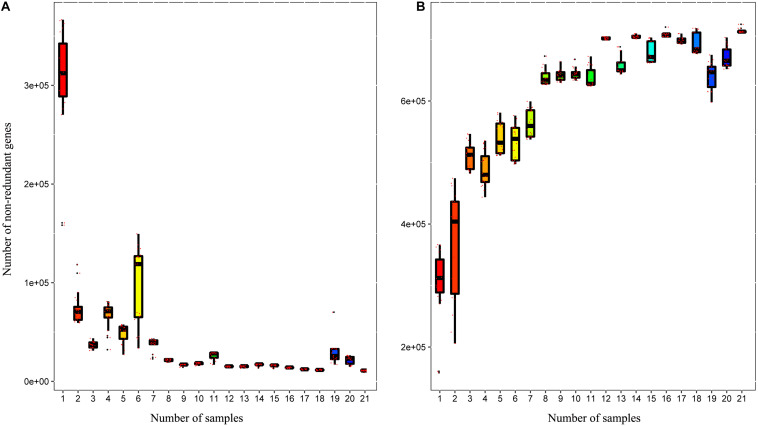
Rarefaction curves of **(A)** core genes and **(B)** pan genes. The horizontal axis represents the number of samples selected randomly, whereas the vertical axis represents the number of genes in the selected samples.

**FIGURE 3:**
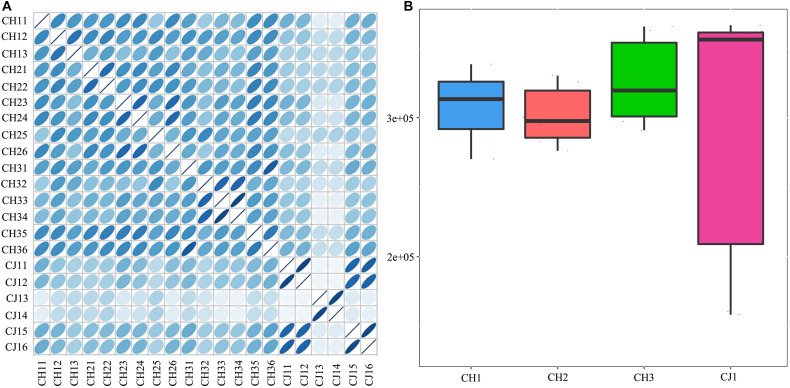
**(A)** Correlation heatmap based on Spearman’s rank correlation coefficient. **(B)** Comparisons of the number of genes among the four groups.

A total of 3,310,621 ORF sequences were obtained, with an average length of 681.09 bp and a GC abundance of 43.39% (Supplementary Table S3). To assess whether the collected samples could meet the requirements of the subsequent bioinformatics analysis, we performed a rarefaction curve analysis. The rarefaction curves based on the Core-Pan genes (Figure 2) gradually became flat and almost reached a plateau, indicating that our gene catalog captured all the genomic content available in our samples. The correlation heatmap based on the Spearman’s rank correlation coefficient (Figure 3A) showed that the difference between the groups was greater than the difference within the groups, indicating reasonable sample selection and reliable experimentation. From the box-plot diagram, we found that the number of genes was higher in the non-estrus period compared to those in the estrus period. More genes were also found in the samples from the south of China (CJ1) than those from the north of China (CH2) (CJ1 > CH3 > CH1 > CH2; Figure 3B).

### Taxonomic Composition

To investigate the relationship between the diversity of fecal microbiota and the estrus period, we profiled the structure of 21 fecal samples using shotgun metagenome sequencing. From the taxonomic annotation of metagenomic data, the majority of the microbiome was bacterial (>98%) with great phylogenetic diversity. The abundances of viruses, Archaea, and Eukaryota were less than 1%.

**FIGURE 4:**
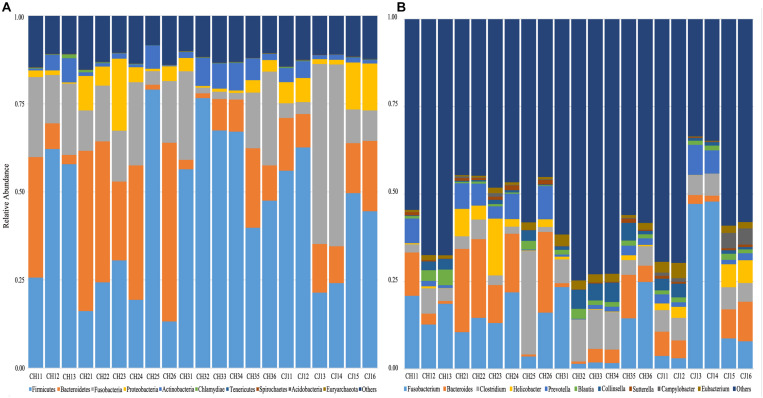
Taxonomic composition of the fecal bacterial communities on the **(A)** phylum and **(B)** genus levels from estrus and non-estrus groups. Each bar represents the 10 most abundant taxa.

**FIGURE 5:**
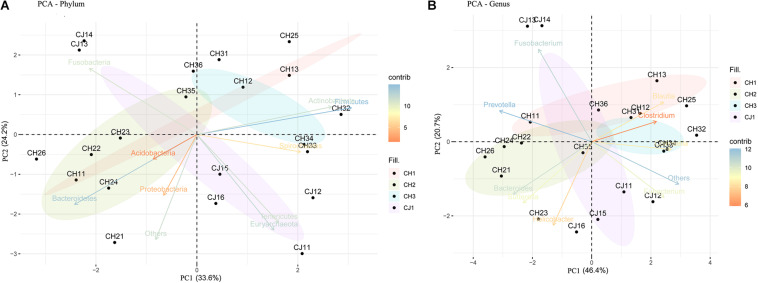
Principal component analysis (PCA) of the fecal bacterial communities on the **(A)** phylum and **(B)** genus levels from estrus and non-estrus groups, showing separation among the groups.

At the phylum level, the sequenced reads were assigned to 31 different phyla and 57 candidates. Firmicutes (44.86% ± 20.90), Bacteroidetes (18.08% ± 15.10), and Fusobacteria (16.44% ± 14.13) were the three dominant phyla in all samples (Figure 4A). However, the dominant phylum differed markedly in each group, and each group had a specific relative abundance of one phylum or specific ratios of two phyla (Supplementary Table S4). In CH2, the relative abundance of *Bacteroidetes* was the highest compared with the other groups (*p* < 0.05). The Firmicutes/Fusobacteria ratio was the highest in CH3 (*p* < 0.05). When we performed hypothesis-testing using Metastat, with *q* < 0.05, only CH2 and CH3 showed significant differences, in the following phyla: Spirochaetes, Synergistetes, Candidatus-Handelsmanbacteria, and Deinococcus-Thermus. DCA showed that the maximum of the first four axes was less than three; thus, PCA was more reasonable. PCA ordination showed separation among the groups (Figure 5A). No clustering was observed within the populations among the groups, except for CH2 and CH3 (ANOSIM: *p* = 0.018, *R* = 0.4685). From a *T*-test, there were significant differences in the relative abundances of Proteobacteria in CH1 and CH2 (*p* < 0.05) and in those of Firmicutes and Bacteroidetes (*p* < 0.05) in CH2 and CH3.

At the genus level, 1,145 genera were identified, with the top 10 genera as follows: *Fusobacterium, Bacteroides, Clostridium, Helicobacter, Prevotella, Blautia, Collinsella, Sutterella, Campylobacter*, and *Eubacterium* (Figure 4B). According to hypothesis-testing using Metastat, with *q* < 0.05, only CH2 and CH3 showed significant differences in the families Erysipelotrichaceae and Lachnospiraceae, including *Allobaculum, Holdemania, Erysipelothrix, Erysipelatoclostridium, Solobacterium, Coprobacillus, Holdemanella Lachnoclostridium, Marvinbryantia*, and *Butyrivibrio.* These were less abundant in the microbiota of CH2 than in CH3 (*q* < 0.05) (Supplementary Table S5). To assess levels of variation, we determined the α-diversity and β-diversity indices at the genus level (Supplementary Table S6). According to the ace and Chao1 indices, we found that the community richness increased from estrus to non-estrus (CH2 to CH3): CH1 > CH3 > CH2 > CJ1. According to the Shannon and Gini-Simpson indices, the community diversity increased from non-estrus to estrus (CH2 to CH3): Shannon, CH1 > CH3 > CH2 > CJ1; Gini-Simpson, CH1 > CH2 > CH3 > CJ1. PCA analysis (Figure 5B) at this level based on significant differences showed that CH2 and CH3 have distinct cluster regions (ANOSIM: *p* = 0.013, *R* = 0.5040).

**FIGURE 6:**
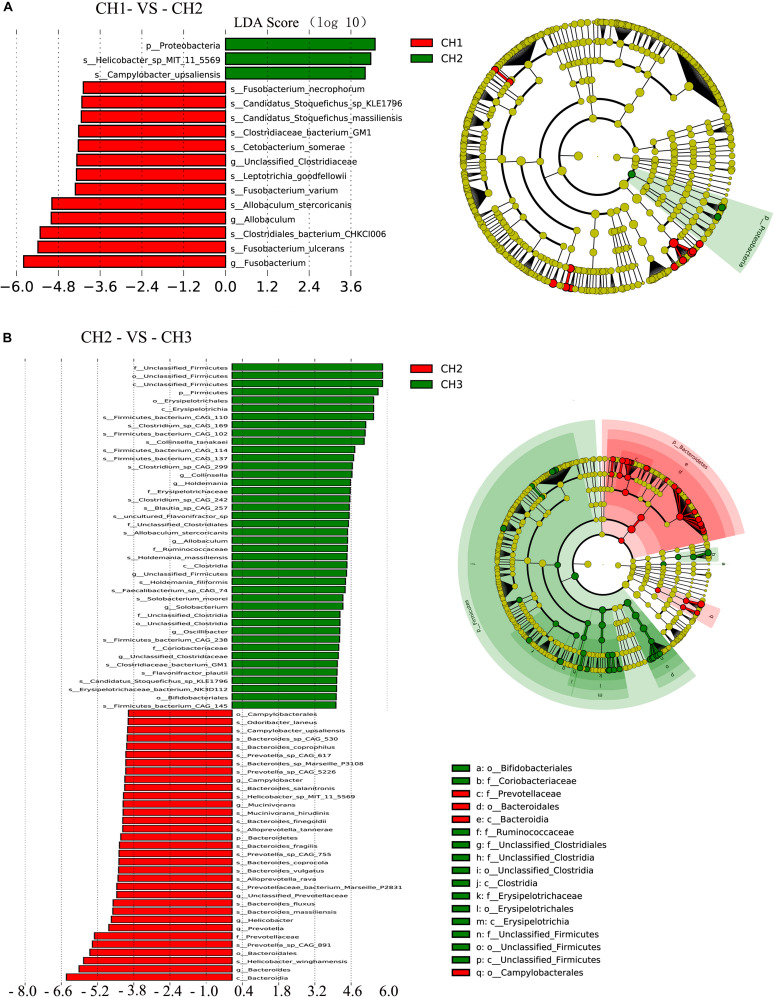
Linear discriminant analysis (LDA) for effect size analysis. The histogram of the LDA score (left) shows the biomarkers with significant differences between the groups. The degree of influence of species is represented by the length of the bar. In the right of the cladogram, the circle radiating inside-out demonstrates the classification (from phylum to genus). Each small circle at a different classification represents a taxon, and the diameter of the circle is proportional to the relative abundance. The species without significant differences are in yellow and biomarkers were colored by different groups. Red and green dots represent the core bacterial populations in the groups. **(A)** CH1 vs. CH2 and **(B)** CH2 vs. CH3.

To assess the differences in the microbial communities as affected by the estrus period, we applied the LEfSe method with an LDA Score > 4. There was a significant difference in abundance among the four groups (Figure 6). Age (CH1 and CH2) may have induced major shifts in microbiota composition. Significant increases in the proportions of *Proteobacteria* at the phylum level were observed with age, particularly Helicobacter-sp-MIT-11-5569, and *Campylobacter upsaliensis*. However, significant decreases were observed in *Unclassified-Clostridiaceae* and *Allobaculum* at the genus level and *Fusobacterium-ulcerans, Clostridiales-bacterium-CHKCI006, Allobaculum-stercoricanis, Fusobacterium-stercoricanis, Leptotrichia-goodfellowii, Cetobacterium-somerae, Clostridiaceae-bacterium-GM1, Candidatus-Stoquefichus-massiliensis, Candidatus-stoquefichus- sp-KLE1796*, and *Fusobacterium-necrophorum* at the species level.

Estrus (CH2 and CH3) induced major shifts in microbiota composition, with significant increases in the proportions of Firmicutes at the phylum level and *Collinsella, Holdemania, Allobaculum, Unclassified-Firmicutes, Solobacterium, Oscillibacter*, and *Unclassified-Clostridiaceae* at the genus level. There were significant decreases in Bacteroidetes at the phylum level and *Campylobacter, Mucinivorans*, and *Unclassified-Prevotellaceae* at the genus level. Other biomarkers of the groups are shown in Figure 6.

### Composition of Metagenome Development

**FIGURE 7:**
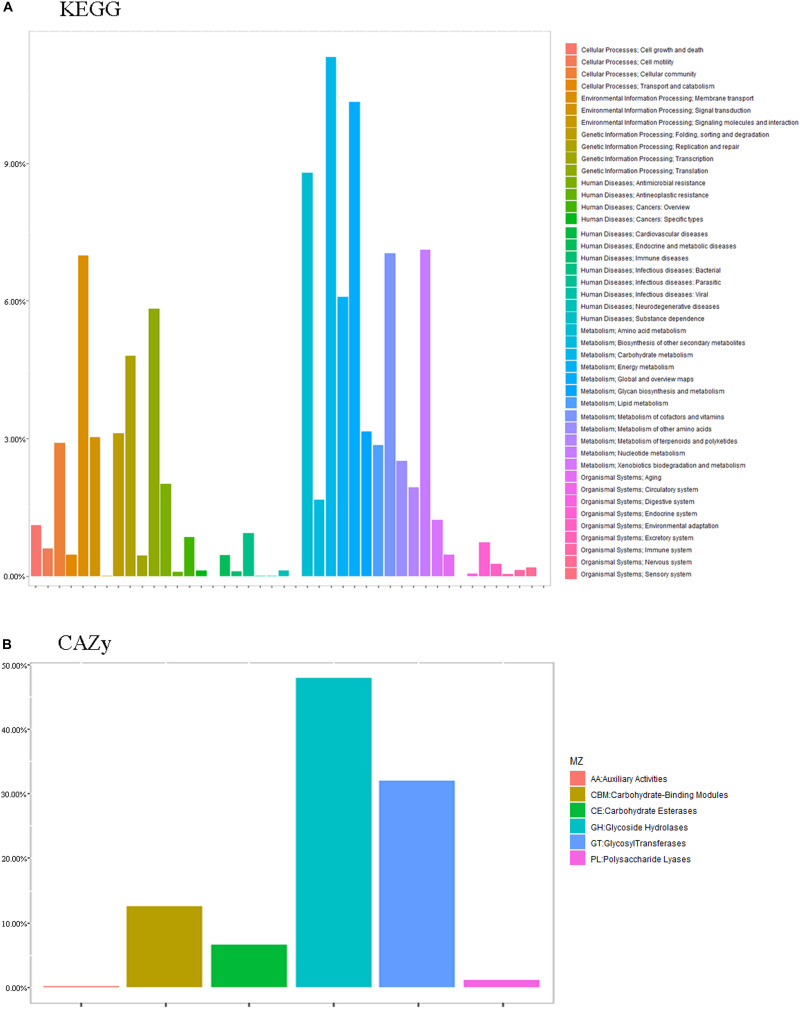
Common functional database annotations. **(A)** KEGG annotations and **(B)** CAZy annotations.

The essence of the host-microbiome interaction is the activity of genes in the microbiome. Thus, we annotated the metabolic and functional pathways of the metagenome based on the CAZy and KEGG databases (Figure 7).

Functional enrichment analysis of the unique genes identified 1,719 enzyme commission (EC) and 4,986 KEGG orthologous (KO) groups whose proportional representation in the fecal microbiomes followed the KEGG database. A large number of the genes were annotated to metabolism (52.28%), including carbohydrate (11.32%), global and overview maps (10.34%), amino acid (8.80%), nucleotide (7.11%), vitamins (7.04%), and energy (6.09%). Also represented were genetic information processing (19.48%), including translation (5.83%), replication and repair (4.80%), and folding, sorting, and degradation (3.12%), and environmental information processing, including membrane (13.53%) and signal transduction (6.71%) (Figure 7).

Only CH2 and CH3 showed significant differences in 11 different pathways at level 2 (*q* < 0.05), including cellular processes-cell growth and death (CH2: 0.42%; CH3: 0.39%), cellular processes-cellular community (CH2: 0.82%; CH3: 1.02%), cellular processes-transport and catabolism (CH2: 0.19%; CH3: 0.09%), environmental information processing-membrane transport (CH2: 2.09%; CH3: 2.54%), metabolism-biosynthesis of other secondary metabolites (CH2: 0.54%; CH3: 0.50%), metabolism-energy metabolism (CH2: 2.31%; CH3: 2.14%), metabolism-glycan biosynthesis and metabolism (CH2: 1.21%; CH3: 0.93%), human diseases-neurodegenerative diseases (CH2: 0.06%; CH3: 0.05%), and human diseases-infectious diseases-bacterial (CH2: 0.34%; CH3: 0.30%). Some pathways with higher abundance in CH2 than in CH3 include cationic antimicrobial peptide (CAMP) resistance (ko01503) and insulin signaling pathway (ko04211). In terms of orthologous genes, the KO results of KEGG showed genes with significant differences from CH2 to CH3 (*q* < 0.01). Some KOs with higher abundance in CH2 than in CH3 include ribonuclease HIII (EC: 3.1.26.4) (K03471), aldehyde: ferredoxin oxidoreductase (EC:1.2.7.5) (K03738), putative lysine transport system permease protein (K17074), tungstate transport system substrate-binding protein (K05772), and putative lysine transport system ATP-binding protein (EC:3.6.3.-). Those with higher abundance in CH3 than in CH2 include 4-hydroxy-tetrahydrodipicolinate reductase (EC:1.17.1.8) (K00215), CTP synthase (EC:6.3.4.2) (K01937), and ATP-dependent RNA helicase dead (EC:3.6.4.13) (K05592).

Functional enrichment analysis of the unique genes identified 431 Enzyme Commission (ECs) whose proportional representation in the fecal microbiome followed the CAZy database. Most of the genes were annotated to the three functional configurations at the second level of CAZy classification: glycoside hydrolases (GH), glycosyl transferases (GT), carbohydrate-binding modules (CBM), and carbohydrate esterases (Figure 7).

**FIGURE 8:**
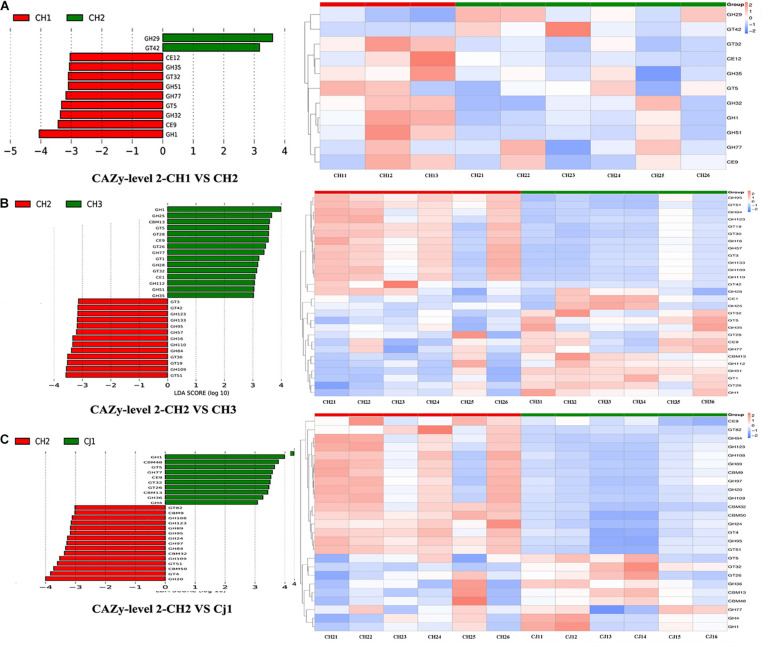
Linear discriminant analysis (LDA) for effect size analysis of the metagenome annotations. The histogram of the LDA score (left) shows the biomarkers with significant differences between the groups. The influencing degree of species is represented by the length of the bar. The heatmap of the corresponding biomarkers (right) shows **(A)** CAZy-level 2-CH1 vs. CH2, **(B)** CAZy-level 2 CH2 vs. CH3, and **(C)** CAZy-level 2 CH2 vs. CJ1.

We also used Metastat to test for significance. At level 2, we found that CH2 and CH3 have more significant differences in the enzyme family subfamily, which is consistent with our previous analysis. Among families, 12 were significantly more abundant in CH2 than in CH3 (*p* < 0.05), including CBM13/23/27/37, CE14/15, GH1/51, and GT1/5/26/87. However, there were more enzymes in CH3 from the GH family. The GH family (>75%) in the CH2 group was significantly greater than that in the CJ1 group. PCA and NMDS were performed on the different units of all database comparisons. The results were still divided into three large clusters: CH1 and CH3, CH2, and CJ1. To screen the functional biomarker with significant differences between the groups, we performed LDA dimensional reduction at all levels of the database and evaluated the impact of the differences in function (Figure 8).

To find significantly different genes between the groups, we performed a PCA analysis based on the functional abundance of the different databases at each classification level. Most of the PCA results showed that all samples can be divided into three groups, namely CH1/3, CH2, and CJ1. The results of the PCoA based on Bray-Curtis distance were consistent with the PCA results. ANOSIM analysis was performed based on the functional abundance of the KEGG-based KO and CAZy’s Level 2. In all ANOSIM analyses, there was a significant difference between CH2 and CH3 (*p* < 0.05). According to the annotated results of CAZy, we found significantly different functional configurations at the second level between CH2 and CJ1 (*p* < 0.001). To study the significant differences between groups, the Metastat method was used for the hypothesis testing of the functional abundance data starting from the functional relative abundance tables at different levels and the abundance box-distribution (Supplementary Table S7).

## Discussion

In this study, we investigated the underlying regulation mechanisms of estrus using metagenomic sequencing of fecal microbiota. Through metagenomic sequencing, we found that the fecal metagenome of dholes contained abundant microbial genes. The estrus period has a significant influence on the fecal microbiome in dholes, suggesting that particular metagenome profiles may promote estrus in this species. In addition, the fecal microbiome of juvenile dholes was similar to that of adult dholes in estrus.

During estrus, dholes were more neurotic—always screaming to attract the attention of the opposite sex and show a decline in appetite (Dalamaga et al., 2013). As the excitement from mating weans, blood sugar levels initially decrease, followed by a decrease in insulin secretion. Lastly, leptin secretion decreases, corresponding to anorexia. In order to explore the influence of estrus on the intestinal microbes, we selected the CH2 and CH3 groups for comparison. For the metagenome dataset of dholes, the alignment-free algorithms (Mash) showed the estrus and non-estrus clustered into two groups, despite the presence of one sporadic data point. From PCA, significant differences in microbial structure and metagenomics were identified in the CH2 and CH3 groups. Regarding bacterial community structures, *Bacteroidetes* was significantly more abundant in the CH2 group. In fact, the abundance of *Bacteroidetes* in the estrus period was lower than that of other normal dholes. Dholes belong to the order Carnivora. However, our analysis of canine feces such as wolves and dholes in the wild revealed the frequent presence of some plant foods such as grass roots and bark in the feces. Thus, we hypothesize that dholes eat a small amount of plants in the wild. At the same time, some plant foods such as corn and sugar cane is added as an indispensable supplement during the feeding process (Zhiyong et al., 2018). A carnivorous animal such as the dhole does not digest polysaccharides found in plant fiber well but we found a high abundance of *Bacteroidetes* in its intestinal microbiota, which can produce a series of enzymes that digest these polysaccharides to provide energy to the host (Wostmann et al., 1983; Younes et al., 2001; Lópezmondéjar et al., 2016; Xiong et al., 2018).

From above analysis, we think there is a potential correlation between gut microbiome and estrus. We will explore the possible impact of altered gut microbes on estrus in the future. Massive secretion of sex hormones occurs during estrus (Fitzpatrick et al., 2014), and beneficial cholesterol is a precursor for the synthesis of sex hormones (Chen, 2013). Here, we found that some bacteria have a positive effect on the production of sex hormones. *Coprobacillus* and *Holdemania* can produce beneficial cholesterol (Kageyama and Benno, 2013; Mishra et al., 2013) and were more abundant in CH3, which were dholes in estrus. Greater concentration of more beneficial cholesterol can induce the increased synthesis of sex hormones, which subsequently leads to estrus. At the same time, estrus can also lead to a dramatic increase in the number of reproductive cells and the synthesis of nucleic acids (Becker, 2018). The genes involved in pathways related to ribonucleases were increased in CH3, which can assist the host in synthesizing a large number of nucleic acids.

After estrus, we observed that dholes were excited and showed weak appetite. The continuous excitement of the body during estrus consumes a lot of energy, with carbohydrate as the direct source (Jéquier, 1994). *Erysipelotrichaceae* can characterize and boost excitability (Myer et al., 2015). The abundance of *Erysipelotrichaceae* in CH3 was significantly higher than that in CH2, indicating that the gut microbes during estrus also characterize the excited state-estrus. Dholes in estrus showed anorexia and the continuous excitement leads to increased consumption of sugar, which lowers the blood sugar level. Therefore, the insulin pathway in the gut microbes of the dhole (CH3) during estrus is inhibited. ferredoxin oxidoreductase (EC: 1.2.7.5) and the lysine transport system ATP-binding protein (EC: 3.6.3.-) in the microbiome of CH3 group increased to produce more energy. This led to an increase in the receptor for acetyl-CoA, which increased the efficiency of glucose metabolism. Environmental Information Processing-Membrane Transport Other was significant, being overrepresented in the CH3 group (20%). The increased activity of this pathway ensures that the fecal microbiome is more positively linked to the host during estrus. In terms of enzyme metabolism, most of the significant differences between the CH2 and CH3 groups involved hydrolysis and/or rearrangement of glycosidic bonds, which were more abundant in CH3. Hydrolysis and/or rearrangement of glycosidic bonds represent the consumption of sugar. Thus, the fecal microbiome can assist the dholes in estrous to meet the vigorous energy demand required during estrus.

Taken together, we elucidated microbiota structures and functions related to estrus. The microbiota may promote the occurrence of estrus, as estrus can affect the intestinal microbes, and these altered intestinal microbes can support the occurrence of estrus.

The different bacterial abundance and pathways were also found in other groups. Through dimensionality reduction analysis, we found that CH1 and CH3 were always grouped together, and there were no significant differences between them. One study revealed that most of the fecal microbiome in infancy is inherited from their pregnant mothers (Bäckhed et al., 2015). Here, we showed that the fecal microbiome of young dholes (CH1) is similar to that in mature dholes in estrus (CH3) but is significantly different from that in mature non-estrus dholes (CH2).

CH2 and CJ1 were studied to produce a more distinct separation. ANOSIM analysis of level 2 of the CAZy showed a significant difference (*p* = 0.003) between these groups. Jiangxi Province has a subtropical warm and humid monsoon climate with an average annual temperature range of 8.6–20.6°C. In contrast, located in the northern coast of China, the Hebei Province has a temperate continental monsoon climate with four distinct seasons with an annual average temperature range of −1.5 and 15.2°C. The climates in these two regions are significantly different, and the dholes raised there also showed significant differences. This is consistent with previous studies showing that climate can affect the fecal microbiome (Eichner et al., 1999; Bäckhed et al., 2004; Spor et al., 2011; Sun et al., 2016). However, because the dholes were excessively stressed during pregnancy, we were not able to collect fecal samples from pregnant females. To better understand the intestinal metagenome of dholes, all life stages should be analyzed.

## Data Availability Statement

The datasets generated for this study can be found in the online repositories. The names of the repository/repositories and accession number(s) can be found in the article/Supplementary Material.

## Author Contributions

HHZ and XYW designed the study and prepared the manuscript. XYW, YQS, QGW, JC, and HXZ conducted the research. XDG and ZYW collected the samples. XYW, QGW, and YC analyzed the data. All authors contributed to the article and approved the submitted version.

## Conflict of Interest

The authors declare that the research was conducted in the absence of any commercial or financial relationships that could be construed as a potential conflict of interest.

## Footnotes

1 https://github.com/cjfields/readfq

2 https://www.ncbi.nlm.nih.gov/sra/SRX1386920

3 https://sourmash.readthedocs.io/en/latest/index.html

4 https://github.com/bbuchfink/diamond/

5 http://www.kegg.jp/kegg/

6 http://www.cazy.org/
